# A Novel Artificially Humanized Anti-Cripto-1 Antibody Suppressing Cancer Cell Growth

**DOI:** 10.3390/ijms22041709

**Published:** 2021-02-08

**Authors:** Hiroko Ishii, Maram H. Zahra, Atushi Takayanagi, Masaharu Seno

**Affiliations:** 1 GSP Enterprise, Inc., 1-4-38 12F Minato-machi, Naniwaku, Osaka 556-0017, Japan; ishii-gsp@tomoikebio.com (H.I.); imai@tomoikebio.com (A.T.); 2 Graduate School of Interdisciplinary Science and Engineering in Health Systems, Okayama University, Okayama 700-8530, Japan; maram@okayama-u.ac.jp

**Keywords:** phage display library, artificial humanized antibody, Cripto-1, anti-Cripto-1 antibody, tissue-micro array, cell growth inhibition

## Abstract

Cripto-1 is a member of the EGF-CFC/FRL1/Cryptic family and is involved in embryonic development and carcinogenesis. We designed a novel anti-Cripto-1 artificial antibody and assessed the recognition to the antigen and the potential to suppress the growth of cancer stem cells. First, single chain antibody clones were isolated by bio-panning with the affinity to recombinant Cripto-1 protein from our original phage-display library. Then, the variable regions of heavy chain VH and light chain VL in each clone were fused to constant regions of heavy chain CH and light chain CL regions respectively. These fused genes were expressed in ExpiCHO-S cells to produce artificial humanized antibodies against Cripto-1. After evaluation of the expression levels, one clone was selected and the anti-Cripto-1 antibody was produced and purified. The purified antibody showed affinity to recombinant Cripto-1 at 1.1 pmol and immunoreactivity to cancer tissues and cell lines. The antibody was available to detect the immunoreactivity in tissue microarrays of malignant tumors as well as in Cripto-1 overexpressing cells. Simultaneously, the antibody exhibited the potential to suppress the growth of human colon cancer derived GEO cells overexpressing Cripto-1 with IC_50_ at approximately 110 nM. The artificially humanized antibody is proposed to be a good candidate to target cancer cells overexpressing Cripto-1.

## 1. Introduction

Phage display was first developed by Smith et al. as an efficient method to select peptides that bind to a target molecule in 1985 [1,2]. In this method, the target peptides are designed to fuse with the coat protein displayed as the outer shell protein of filamentous bacteriophage, typically M13 infected in host bacteria with amber mutation. The phage display method has long been applied to isolate polypeptides with desired functions in various fields such as antibody engineering.

Anticancer drug treatment has made great progress since when the first antibody drug was approved and launched in Japan in 2000. However, enormous efforts and huge cost have been required in the development and production of antibodies by preparing antigens, immunizing animals and selecting the best antibody from among the candidates. Winter et al. successfully developed the phage display technology as a simple and in vitro method to discover antibodies [3,4]. The phage display technology is currently applied in many studies of drug discovery. For this achievement, Smith and Winter were awarded the Nobel Prize in Chemistry in 2018 [5].

Cripto-1 (CR-1), a member of the EGF-CFC/FRL1/Cryptic family, is a GPI-anchored protein functioning as a coreceptor of Nodal, which is a member of TGF-beta family mediating ALK4/Smad2 signaling maintaining CSCs [6]. Expression of CR-1 is observed in early embryogenesis and often in the development of many cancers. However, due to the lack of elucidation of the function of CR-1, studies focusing on CR-1 as a target of cancer therapy was limited for a long time. However, in recent years, the function of CR-1 in cancer cells has been described and suggested to be a good target of cancer treatment [7,8,9]. The expression level of CR-1 is enhanced in various tumors supporting cancer cell proliferation, migration, epithelial–mesenchymal transition and stimulation of tumor angiogenesis, while it is very low in normal adult tissues [10]. Sandomenico et al. reviewed Nodal, Cripto-1 and the complexes as the target on the surface of tumor cells, especially cancer stem cells (CSCs) [11]. Targeting these biomarkers will lead to the development of potential antitumor agents that overcome both drug resistance and recurrence. In a recent study, Daraghma et al. showed the potential of co-targeting the Nodal and CR-1 proteins for the treatment of oral squamous cell carcinoma [12]. Alowaidi et al. investigated the effect of CR-1 on pathways that control glioblastoma cells in phosphorylation-specific protein microarray analysis. They also suggested that angiogenesis may be mediated by Cripto-1, which regulated the motility and infiltration of cancer cells [13].

Here, in this study, we tried to establish an anti-CR-1 antibody using our original single-chain Fv antibody (scFv) phage display library [14], which was designed with the fused variable regions of heavy and light chains coded in human antibody genes. The isolated phage clones with the affinity to CR-1 protein were assessed for the potential of targeting cancer cells and suppressing the cell growth.

## 2. Results and Discussion

### 2.1. Production of Humanized Anti-Human CR-1 Artificial Antibody

**Figure 1:**
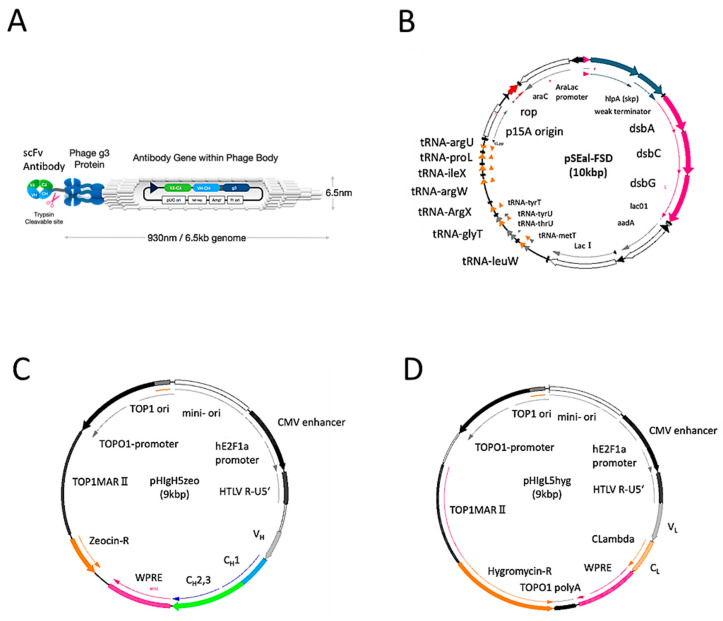
Vector information of phage library and Human IgG: (**A**) schematic structure of a phage displaying scFv; (**B**) the structure of plasmid pSEaL-FSD4 harbored in E. coli XL1Blue; (**C**) the structure of plasmid *pHIgH5zeo* conveying the DNA sequences for human IgG V_H_ region followed by C_H_ region consisting of C_H_1, C_H_2 and C_H_3 with a zeocin resistant gene; and (**D**) the structure of plasmid *pHIgL5hyg* conveying the DNA sequences for human IgG V_L_ region followed by C_L_ with a hygromycin resistant gene. See text for more details.

**Figure 2:**
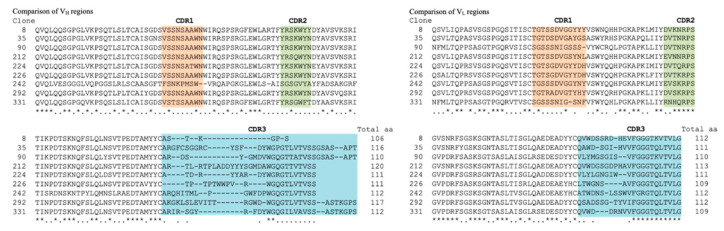
Comparison of the amino acid sequences of V_H_ and V_L_ regions in the nine clones isolated from the phage library by bio-panning. The domains corresponding to CDR1, CDR 2 and CDR 3 are highlighted with each colored background.

We used the original scFv phage library consisting of M13 derived phagemid and chaperon coding plasmid (Figure 1A,B). With human CR-1 as an antigen the library was screened and the cDNAs of V_H_ and V_L_ in the isolated clones were expressed in the artificial antibody expression vectors (Figure 1C,D). As a result, nine phagemid clones recognizing CR-1 were isolated by affinity bio-panning. The insert DNAs in the selected nine phagemid clones were sequenced and translated into amino-acid sequences (Figure 2). The sequences corresponding to CDR1, CDR2 and CDR3 in both V_H_ and V_L_ were compared. The amino acid sequences of V_H_ ranged from 106 to 117 residues and those of V_L_ from 109 to 113. In both V_H_ and V_L_, CDR1 and CDR2 regions were consisting of very similar amino acid sequence, respectively. Only the critical differences were found in CDR3 regions. However, the core sequences appear as WGP/QGTLVTVSS in V_H_ and FGGGTQVTVLG in V_L_. These clones were found to encode similar structure of variable regions recognizing human CR-1. The V_H_ and V_L_ sequences from the nine clones were subsequently designed as humanized artificial antibody against human CR-1 with the original vectors *pHIgH5zeo* and *pHIgL5hyg*. These two vectors were designed to express artificially humanized anti-human CR-1 V_H_-C_H1_-C_H2_-C_H3_ and V_L_-C_L_ as anti-CR-1 Ab and transfected to ExpiCHO-S cells.

**Figure 3:**
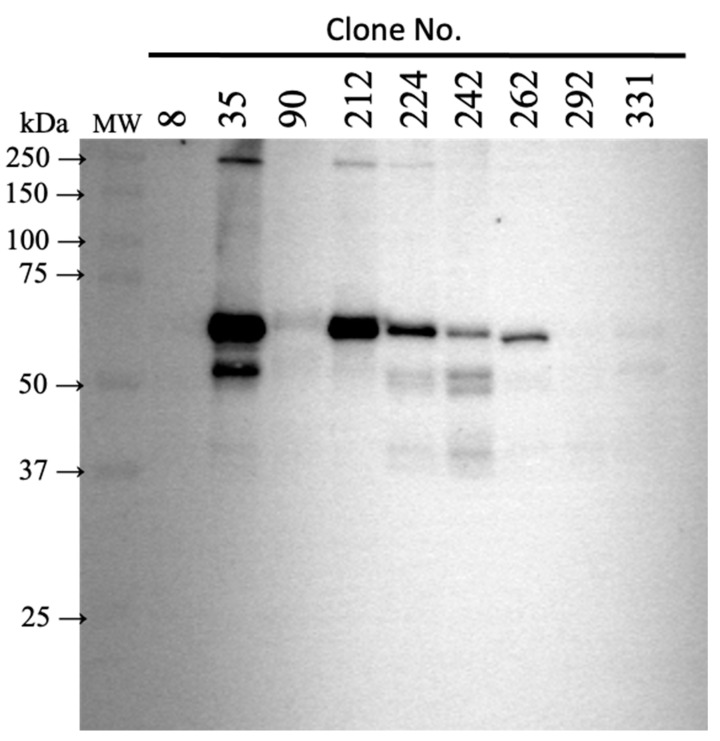
Expression of humanized IgG clones. The conditioned media were analyzed by Western blotting with anti-human IgG-Fc antibody. The conditioned medium of non-transformed ExpiCHO-S cells was used as a negative control.

We assessed the binding ability in the conditioned medium of ExpiCHO-S cells to rhsCR-1 fixed on the AR2G sensor of OCTET system. These results indicate that the anti-CR-1 Abs in the conditioned medium had affinity for the antigen (Table S1). Then, IgG in the conditioned medium was confirmed by Western blotting (Figure 3). Since the detected IgG expression level was the highest in the Clone 35, further characterization of the antibody was performed with Clone 35.

### 2.2. Physicochemical Characterization of Artificial Antibody

**Figure 4:**
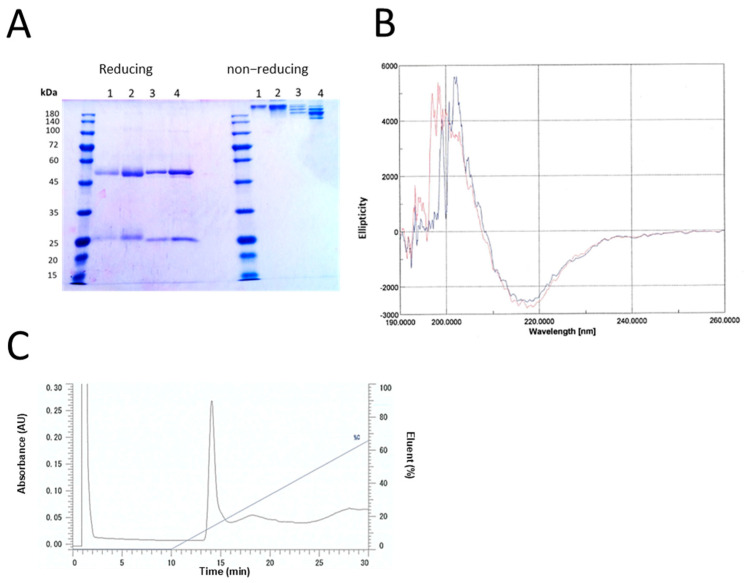
Physicochemical characterization of anti-CR-1 antibody Clone 35. (**A**) purified anti-CR-1 antibody to CR-1 was analyzed by SDS-PAGE under reducing and non-reducing conditions. Lanes 1–2, human-IgG; Lane 1, 1 µg; Lane 2, 2 µg; Lanes 3–4, purified anti-CR-1 antibody (reduce or native form); Lane 3, 1 µg; Lane 4, 2 µg. (**B**) CD spectra of anti-CR-1 antibody Clone 35 (red) and human IgG (blue). The range of wavelength is between 190 and 260 nm. Profiles are depicted as the accumulation of four times. (**C**) The elution profile of anti-CR-1 antibody Clone 35 from anion column under the NaCl gradient of 0 to 1 M (100%) monitored at 280 nm.

Anti-CR-1 antibody derived from the phage Clone 35 was purified from the conditioned medium of ExpiCHO-S cells and assessed by SDS-PAGE under reducing and non-reducing conditions (Figure 4A). The yield of full-length antibody secreted in the conditioned medium of ExpiCHO-S cells was estimated as 2 mg/L of culture. Under reducing condition, anti-CR-1 antibody Clone 35 exhibited two bands of 50 and 25 kDa, which were equivalent to the sizes of heavy chain and light chain of human IgG, respectively, while high molecular weight of protein bands were recognized under non-reducing condition. The profiles of the secondary structure of the antibody were analyzed by circular dichroism (CD) spectra (Figure 4B). The patterns of regular and irregular structures were compared with human IgG to estimate the contents of α-helix, β-structure and β-turn. The results show that anti-CR-1 antibody Clone 35 has the secondary structure very similar to human IgG antibody. Subsequently, we assessed the elution profile of anti-CR-1 Clone 35 antibody using anion exchange column liquid chromatography (Figure 4C). Anti-CR-1 Clone 35 antibody was eluted in one peak in the chromatogram, which meant the structure of the antibody was homogeneous. Collectively, anti-CR-1 antibody Clone 35 was concluded to be purified in a single population of protein and to have the structure correctly folded as an artificial human IgG.

### 2.3. Evaluation of the Affinity of Humanized Artificial Antibody to Antigen

**Figure 5:**
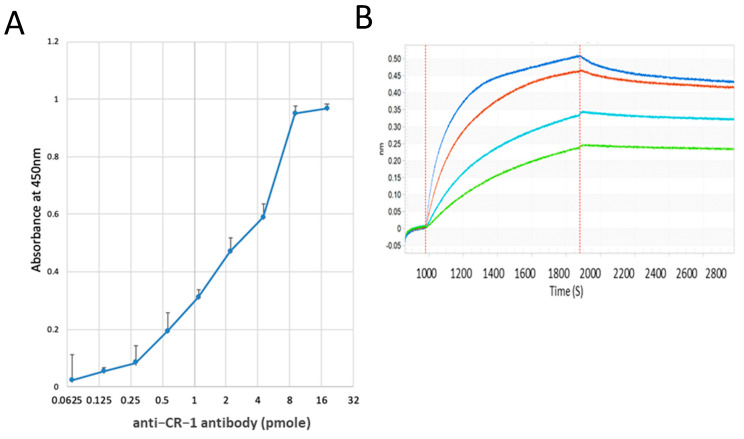
Affinity of anti-CR-1 antibody Clone 35 to antigen: (**A**) The binding ability of purified anti-CR-1 antibody to CR-1 was assessed by ELISA. Anti-CR-1 antibody in 0.68 mg/mL was sequentially diluted and applied into 96-well plate, which was fixed with rshCR-1. The bound antibody was detected with anti-human IgG labeled with HRP. Each plot was shown as mean ± SD from three independent experiments. (**B**) The global fitting curves demonstrating the affinity of anti-CR-1 Ab to rshCR-1 analyzed by BLI method. Each concentration of antibody is 25 nM (green), 50 nM (light blue), 100 nm (brown) and 200 nM (blue).

Anti-CR-1 antibody was sequentially diluted in two-fold starting from 18 pmol/well (0.68 mg/mL) in an ELISA plate, which was prepared with rshCR-1 at 0.5 µg/well in advance. As a result, 1.1 pmol of anti-CR-1 antibody was found to be available to detect 0.5 µg of rshCR-1 (Figure 5A). Simultaneously, the affinity of anti-CR-1 antibody to antigen was assessed by the Octet system (Figure 5B). rshCR-1 (2 ng/mL) fixed to an amine reactive biosensor and assayed with anti-CR-1 antibody in the range between 25 and 200 nM. As a result, all of each result showed the same affinity regardless of the antibody concentration (Table 1). This indicates that anti-CR-1 antibody Clone 35 has affinity to the antigen.

### 2.4. Characteristics of Anti-CR-1 Antibody Clone 35 in Immunostaining

**Figure 6:**
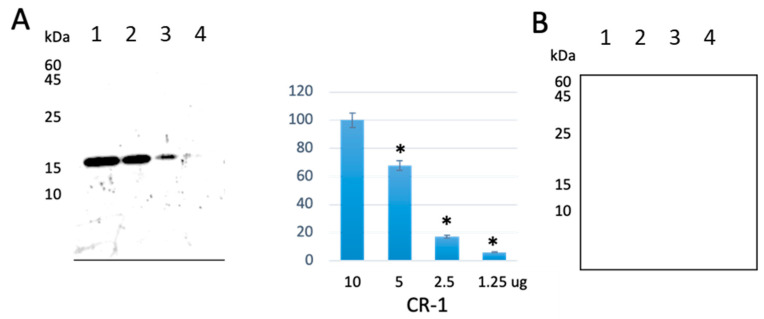
Western blotting of CR-1 antigen detected by anti-CR-1 Ab: (**A**) Reducing condition (left) and densitometric analysis of the band detected on the membrane (right). Each bar is the mean ± SD from three independent experiments. * *p* < 0.05. (**B**) Non-reducing condition. Lane 1, 10 µg; Lane 2, 5 µg; Lane 3, 2.5 µg; Lane 4, 1.25 µg. The primary antibody was anti-CR-1 Ab Clone 35 (500 ng/mL), while the secondary antibody was anti-human IgG HRP (dilution 1:5000).

**Figure 7:**
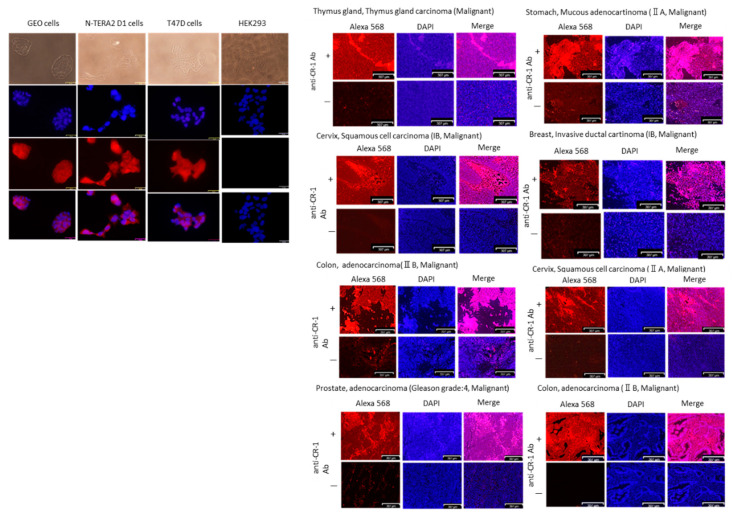
Immunostaining of cancer cell lines and tissues with anti-CR-1 antibody Clone 35: (**A**) Immunoreactivity of anti-CR-1 antibody on cell lines overexpressing CR-1. HEK293 cells are the negative control of CR-1 expression. Scale bars = 32 µm. (**B**) Immunoreactivity of human tissue-microarray with anti-CR-1 antibody. Anti-human IgG conjugated with Alexa 568 was used as the secondary antibody. Negative control was assessed without the primary antibody (Figure S1). Scale bars = 300 µm.

To assess the availability of anti-CR-1 Ab Clone 35 on the immunostaining of cells, we tried Western blotting (Figure 6) and immunocytochemistry (Figure 7 and Figure S1). Anti-CR-1 antibody could detect CR-1 antigen under reducing but not under non-reducing conditions. More than 2 µg (130 pmol) of CR-1 was necessary to be detected even in non-reducing conditions. Taking the results of ELISA (Figure 5A) with high sensitivity into consideration, anti-CR-1 antibody Clone 35 appears to recognize the intact 3D structure of CR-1 in efficient manner more than the structure denatured by SDS. However, the difference observed between the reducing and non-reducing conditions is still not clear. Immunocytochemistry was simultaneously assessed on the cell lines overexpressing CR-1 (Figure 7A). As a result, human cancer cell lines GEO, N-Tera2 D1 and T47D exhibited clear immunoreactivity to anti-CR-1 antibody Clone 35 while not HEK293 cells, which are known to be negative for CR-1 expression. Thus, the affinity of anti-CR-1 Ab Clone 35 to CR-1 protein was successfully demonstrated in immunostaining.

Subsequently, the human cancer tissue array containing thymus gland, prostate, stomach, cervix, colon, breast and prostate was evaluated by the immunostaining with anti-CR-1 antibody Clone 35 (Figure 7B). The cancer tissues were positively and partly stained with anti-CR-1 antibody Clone 35, suggesting many cancer tissues should contain a subpopulation expressing CR-1.

### 2.5. Inhibitory Effect of Anti-CR-1 Ab on Cancer Derived Cells

**Figure 8:**
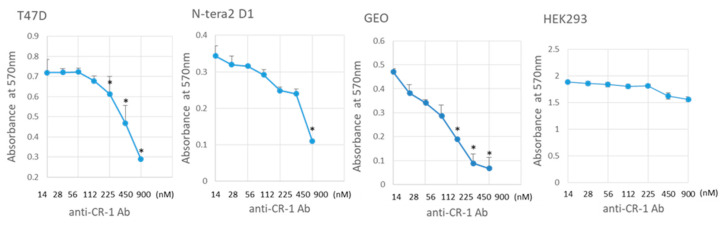
The cell growth inhibitory effect by anti-CR-1 antibody Clone 35. See text for details. Each plot is the mean ± SD. Significance was evaluated with the maximum point. * *p* < 0.05.

With the anti-CR-1 antibody Clone 35, the growth inhibition of CR-1 overexpressing cells, T47D cells, Ntera2 D1 cells and GEO cells was assessed in the range 2.1–136 µg/mL (14–900 nM) (Figure 8). The antibody exhibited the highest growth inhibitory effect on GEO cells among the three cell lines. The IC_50_ of this antibody on GEO cells was approximately 17 µg/mL (110 nM), whereas HEK293 cells did not show any suppression of the growth. This IC_50_ value of anti-CR-1 Ab is comparable to that of cetuximab targeting EGFR, which is 227 nM on the human adenocarcinoma cell line NUGC-4 cells (Genomics of Drug Sensitivity in Cancer, https://www.cancerrxgene.org (accessed on 18 January 2021)). The IC_50_ of antibodies is not always as low as those of chemotherapy because antibody-dependent cellular cytotoxicity (ADCC) and/or complement-dependent cellular cytotoxicity (CDCC) are sometimes significant for antibody medicine. Although the doses of the amount of administration of the antibody should be considered to avoid immunological response when evaluated in vivo, the anti-CR-1 antibody Clone 35 may exhibit the effects of ADCC/CDCC. If CR-1 is taken as a surface biomarker specific to cancer stem cells [15,16,17], targeting CR-1 will be effective to suppress the recurrence and metastasis.

## 3. Materials and Methods

### 3.1. Cell Cultures

As the CR-1 overexpressing cells, human breast carcinoma cell line T47D [18,19] and human teratocarcinoma cell line N-tera2 D1 [20,21] were obtained from American Type Culture Collection (ATCC) (Manassas, VA, USA), and human colon cancer cell line GEO [22,23] was kindly provided by Dr. David Salomon under the MTA of National Cancer Institute, Bethesda, MD. HEK293 cells [24,25] were obtained from ATCC. ExpiCHO-S cells were from Thermo Fisher Scientific (Waltham, MA, USA). T47D, GEO and HEK293 cells were maintained in DMEM with high glucose formula (WAKO, Tokyo, Japan) containing 10% FBS (Thermo Fisher Scientific, Waltham, MA, USA) and 1% penicillin–streptomycin (WAKO, Tokyo, Japan) at 37 °C under the atmosphere of 5% CO_2_. ExpiCHO-S cells were cultured in ExpiCHO Expression Medium (Thermo Fisher scientific, Waltham, MA, USA) at 37 °C under 8% CO_2_ shaking at 120 rpm [26,27].

### 3.2. scFv Phage Display Library

The scFv phage display library used in this paper has previously been described [14]. Briefly, the human cDNAs for variable regions of heavy and light chains were amplified by PCR and cloned into lox containing phagemid vectors independently. Then, the recombination between two lox sites between the phagemids took place during the culture and scFv library was constructed (Figure 1A). The phagemid clones were designed to express the fusion protein of V_H_ and V_L_, which was fused to the amino-terminus of M13 g3 protein flanked by trypsin susceptible amino-acid sequence, KIKG, followed by an amber stop codon, which should be read through when expressed in E. coli with *sup44* upon the infection of the helper phage M13AX1, which was modified from M13KO7 by replacing *p15A* replication origin with *CloDF13* replication origin and inserted into the *rop* gene. In this experiment, the E. coli XL1Blue harboring the plasmid *pSEal-FSD* (Figure 1B), which was composed of periplasmic chaperon and disulfide isomerase genes, *skp*, *fkpA*, *dsbA*, *dsbC* and *dsbG* inducible by IPTG and arabinose, continuously expressing *lacI* and a spectinomycin-resistant genes, rare tRNA genes, *argX*, *argW*, *argU*, *leuW*, *glyT*, *ileX*, *proL*, the rop gene derived from pBR322 and p15A replication origin, was used as the host bacteria. The host bacterial cells were cultured at 37 °C for 16 h in PD medium containing 3% *w*/*v* BACT tryptone (Gibco, Waltham, MA, USA), 2% *w*/*v* yeast extracts (Gibco, Waltham, MA, USA), 0.5% *w*/*v* NaCl, 0.6% *w*/*v* glycerol, 0.05% *w*/*v* MgSO_4_/7H_2_O, 0.02% *w*/*v* K_2_HPO_4_ in 10 mM MOPS-NaOH (Dojin, Kumamoto, Japan) at pH 7 and harvested the supernatant by centrifugation.

### 3.3. Screening for scFv Displaying Phage Clones 

The phages with the affinity to recombinant soluble human CR-1 protein (rshCR-1) [28] were concentrated by bio-panning. After three rounds of bio-panning, phage clones exhibiting the affinity to rshCR-1 were isolated. For the panning, rshCR-1 was covalently immobilized at 1 µg/well in 16 wells of Nunc^®^ Immobilizer™ Amino strip-well plate (Thermo Fisher Scientific, Waltham, MA, USA). Then, unreacted functional groups in each well were saturated with 3% BSA in phosphate buffered saline without Mg^2+^ and Ca^2+^ (PBS). Then, the culture supernatant of the phage library prepared above was serially diluted with PBS in the wells and incubated for 1 h at 25 °C. After washing five times with PBS containing 0.1% Tween 20, the phages bound to antigen were harvested two times by treatment with TrypLE Express (Thermo Fisher Scientific, Waltham, MA, USA) for 5 min. Then, the phages were infected to E. coli XL1Blue harboring pSEal-FSD (Figure 1B) in the presence of 50 µg/mL of spectinomycin (Wako, Tokyo, Japan), for 1 h at 37 °C. Then, helper phage M13AX1 was added and incubated at 25 °C for 5 min. PD medium containing 1 mM IPTG (Wako, Tokyo, Japan), 0.01% *w*/*v* of arabinose (Wako, Tokyo, Japan), 10 ng/mL of anhydrotetracycline (aTet) (Wako, Tokyo, Japan), which induces scFv-g3 fusion protein from the phagemid, was added and E. coli were cultured at 37 °C for 1 h. Then, 15 µg/mL of carbenicillin (Wako, Tokyo, Japan), 50 µg/mL of kanamycin (Wako, Tokyo, Japan) and 12.5 µg/mL of chloramphenicol (Wako, Tokyo, Japan) were supplemented and further cultured at 37 °C with continuous aeration until growth saturation. The amplified phages in the culture supernatant were precipitated with 20% polyethylene glycol containing 2 M NaCl and suspended with PBS for further procedures. In the second panning, wells with immobilized rshCR-1 were blocked with 5% solution of PerfectBlock (MoBiTec, Goettingen, Germany) in PBS. After 1-h incubation with the phages, the wells were washed with 1% of Triton-X100 in PBS 10 times and with PBS containing 1% each of arginine, glutamine, leucine and phenylalanine 10 more times. Then, the phages were harvested and amplified as described above. The third panning was done similarly except for blocking wells with N101 polymer (NOF, Tokyo, Japan). The harvested phages were infected to E. coli XL1Blue and plated to form colonies on LB agar plate containing 100 µg/mL of ampicillin (Wako, Tokyo, Japan) at 37 °C. Single colonies that showed a single band ranging approximately 800 to 1000 bases amplified by PCR with the primers pFab5seq-f (5′- GAT TTG CGC TGT CGC TCC TTG -3′) and pFab5seq-r (5′- CCC AGC CAG GCT TCC ACA TCA C -3′) were selected.

### 3.4. Preparation of Recombinant IgG Protein

The cDNAs cloned in the phages coding variable regions of the heavy chain (V_H_) and the light chain (V_L_) were amplified by PCR, and subcloned into Esp3I sites into our original vectors, *pHIgH5zeo* (Figure 1C) for the heavy chain and *pHIgL5hyg* (Figure 1D) for the light chain. The plasmids were purified with NucleoBond Xtra Midi kits (MACHEREY-NAGEL, Dueren, Germany). The plasmid DNAs were transfected into ExpiCHO-S cells with PEI-MAX (MW 2.5K, Polymer Science, Monticello, IN, USA). In detail, 27 µg of PEI-MAX was gently mixed with 6 µg of DNA (3.0 µg of pCMV-GFP (Addgene, Watertown, MA, USA), 0.75 µg of *pHIgH5zeo* and 2.25 µg of *pHIgL5hyg*) for 30 min at 37 °C [29]. Then, DNA/PEI mixture were mixed with 2 × 10^7^ ExpiCHO-S cells and diluted 4 folds with CH400AZ medium (GMEP, Fukuoka, Japan) supplemented with 0.5 mM valproic acid (WAKO, Tokyo, Japan), 1/10 volumes of the Feed medium (GMEP, Fukuoka, Japan) and 0.5% *w*/*v* glucose together with 0.025% *w*/*v* N,N-dimethylacetamide (Wako, Tokyo, Japan) and 24 µg of shredded salmon sperm DNA (Wako, Tokyo, Japan) and cultured at 32 °C shaking at 130 rpm under the atmosphere of 5% CO_2_. After 4 days, 1/10 volumes of the Feed medium and 0.5% *w*/*v* Glucose were supplemented. Then, the culture supernatant at Day 10 was harvested and passed through 0.22-µm filter (Sartorius, Göttingen, Germany) to apply to HiTrap protein A column (1 mL, GE Healthcare, Chicago, IL, USA). After washing with 20-column volume of PBS, the containing recombinant IgG was eluted with glycine–HCl at pH 3, neutralized with Tris-HCl and pH 9 and then dialyzed against PBS. The amount of protein in the eluted fraction was determined by ProteinAssay (BioRad Labs, Hercules, VA, USA) with human IgG (Wako, Tokyo, Japan) as a control.

### 3.5. OCTET Analysis to Evaluate the Affinity to Recombinant Human CR-1

Bio-layer interferometry (BLI) method was used to evaluate the affinity of IgG to antigen with Octet RED96 (ForteBio, Fremont, CA, USA) [30,31]. Anti-human CR-1 artificial antibodies (anti-CR-1 Abs) derived from these clones in ExpiCHO-S cell culture conditioned medium were analyzed by BLI. The Amine Reactive biosensor chips (AR2G) were activated in WSC/NHS and then rshCR-1 was immobilized on the biosensor by incubating for 90 min. After briefly placing in PBS, the sensor chips were placed in blocking buffer for 30 min and assigned in the Octet system. The layout of wells in plate was set up and assay steps were defined in the Octet data acquisition software. KD, kdis and response were determined by Octet analysis software equipped with the system.

### 3.6. Circular Dichroism

Human IgG (IBL, Gunma, Japan) and anti-CR-1 antibody Clone 35 were adjusted to 1 mg/mL using PBS. The measurements were performed in a room degree using a circular dichroism spectrometer J-720W (JASCO, Tokyo, Japan) and a cell with an optical path length of 1.0 mm. Measurements were also performed at 0.1 nm intervals in the wavelength range 190–260 nm. The protein structure was confirmed by comparing the expressions of increase and decrease of the ellipticity of the average residue at a specific wavelength. Helicity was estimated by curve fitting of the CD spectrum using the determined spectrum [32].

### 3.7. Anion Exchange Column Chromatography

Ten micrograms of anti-CR-1 Ab was analyzed by anion exchange column Inertsil AX column (ø4.6 mm × 150 mm, GL Sciences, Tokyo, Japan). The elution was performed using a linear gradient of NaCl concentration from 0 to 1 M in 10 mM Tris-HCl, pH 8.0, for 30 min at a flow rate of 0.5 mL/min. The absorbance was monitored at 280 nm. The chromatography was performed with the HPLC system D2000 (Hitachi, Tokyo, Japan).

### 3.8. ELISA Assay Detecting Recombinant Human IgG

In a 96-well plate (nerbeplus, Winsen, Germany), 0.5 µg of rshCR-1 in PBS was adsorbed per well by incubation at 4 °C overnight. After washing three times with PBS, wells were blocked with 5% skim milk in PBS containing 1 mM EDTA by the incubation at 25 °C for 1 h. After three subsequent washes with PBS, sequentially diluted recombinant antibodies were added and incubated for 2 h at 25 °C. After treatment with the primary antibody, the plates were washed five times with PBS containing 0.1% Tween 20 (PBST). Then, anti-human IgG goat/rabbit polyclonal antibody conjugated with horse radish peroxidase (HRP) (abcam, Cambridge, UK) was incubated for 2 h at 25 °C. After washing five times with PBST, tetramethylbenzidine (Thermo Fisher Scientific, Waltham, MA, USA) was added at 100 µL/well as the substrate of HRP and incubate for 30 min and the reaction was stopped with the equal volume of 2 N sulfuric acid. The developed color at 450 nm was measured by microplate reader SH-9000 (Hitachi, Tokyo, Japan).

### 3.9. Western Blotting Analyses

The purified recombinant antibodies were applied to SDS-PAGE and electrophoretically transferred to PVDF membrane (Bio-Rad Lab, Hercules, CA, USA). Membranes were blocked with 5% skim milk and incubated at 25 °C for 16 h with anti-human IgG conjugated with HRP (Life Technologies, Carlsbad, CA, USA) diluted to 1/1000. After wash with PBS, immunoreactive bands were developed with ECL detection reagents (Thermo Fisher Scientific, MA, USA) and observed by Light Capture II system (ATTO, Tokyo, Japan).

### 3.10. Fluorescence Staining of Cells

The cells were cultured for over 1 week with medium exchange every 2 days followed by sufficient passage before confluency at 37 °C, 5% CO_2_. The expression of CR-1 was confirmed by immunofluorescent staining. After fixing with 4% paraformaldehyde at room temperature for 20 min, the glass plate was washed 3 times and incubated with 0.1% Triton X-100 in PBS at room temperature for 10 min. After blocking with 2% BSA for 1 h at room temperature, the cells were incubated with anti-CR-1 antibody Clone 35 (dilution 1:25) overnight at 4 °C. After washing 3 times, the secondary antibody, Alexa flour 568 anti-human antibody (A21090, Invitrogen, Carlsbad, CA, USA), was incubated at room temperature for 1 h. Vectashield Hardset (VECTOR, Burlingame CA, USA) was used for DAPI staining.

### 3.11. Immunostaining of Tissue Microarrays

Tissue microarray slides (US Biomax Inc., Rockville, MD, USA) were boiled in 10 mM Na-Citrate, pH 6.5 for 30 min under high pressure [33,34]. The enzymes in the tissue were inactivated with 3% H_2_O_2_ by the incubation for 20 min and the slides were washed 3 times in PBS followed by blocking with 5% skim milk for 40 min at 25 °C. After washing with PBS, the slides were exposed to the recombinant antibody with appropriate dilution in PBS. After incubation for 16 h at 4 °C, anti-human IgG goat IgG conjugated with Alexa 568 (A-21090,Thermo Fisher Scientific, Waltham, MA, USA) diluted to 1/500 was applied to detect the primary antibody followed by staining nuclei with 4,6-diamidino-2-phenylindole (DAPI) (Thermo Fisher Scientific, Waltham, MA, USA). The results of the staining were observed with a fluorescent microscope FSX100 (OLYMPUS, Tokyo, Japan).

### 3.12. Evaluation of Cell Growth Inhibition

Cell growth was assessed by the incorporation of 3-(4, 5-dimethylthiazol-2-yl)-2, 5-diphenyltetrazolium bromide (MTT) (Dojin, Kumamoto, Japan). Briefly, 5 × 10^3^ cells were seeded per well in 96-well plates (Thermo Fisher Scientific, Waltham, MA, USA). After 16 h, sequentially diluted recombinant antibody was added to each well and incubated for 72 h. The cells were then exposed to a final concentration of 1 mg/mL of MTT for 4 h. Precipitated formazan crystals were dissolved with 10% SDS in 0.02 N HCl for 16 h at 37 °C. The absorbance at 570 nm was subsequently measured by the microplate reader [35]. The measurements from six independent experiments were used to calculate the means with standard deviations.

## 4. Conclusions

Phage clones with affinity to human CR-1 displaying sequences of CDR1, CDR2 and CDR3 in both human IgG V_H_ and V_L_ regions were isolated from the original scFv phage display library. From the phage clones, humanized artificial antibodies against human CR-1 were designed, prepared and characterized. Anti-CR-1 antibody Clone 35 recognized CR-1 in intact structure when compared to the denatured structure of the antigen. This antibody successfully exhibited immunoreactivity, immunocytochemistry and immunohistochemistry, suggesting the availability in the pathological diagnosis of cancer tissues. The growth inhibitory effect of the antibody was also demonstrated on CR-1 overexpressing cells. Further investigation targeting CR-1 will lead to the development of agents to treat cancer. However, there are still many issues with this antibody. For example, side effects are often discussed by researchers in cancer treatments. Chemotherapies are well known to exhibit different side effects depending on the targets. On the other hand, many of the side effects of antibody medicines are shown to be boosting immune responses. Probably the side-effects of anti-CR-1 Ab will be the same as the others even if it is found. We will focus this point in the future paper on the experiments in vivo.

## Figures and Tables

**Table 1:** Affinity of anti-CR-1 antibody to rshCR-1 measured by OCTET system.

Antibody Concentration (nM)	Response*^1^ × 10^−1^	Kdis*^2^ × 10^−2^/s
25	9.95	1.14
50	9.95	1.14
100	9.95	1.14
200	9.95	1.14

Each Value from Individual Concentrations is calculated by the Steady State Analysis Section of OCTET System Based on the Global Fitting Curves in Figure 5B. *1. Response Calculated from the Time Window Entered in the Steady State Analysis Section of OCTET System. *2. kdis (1/s) Value Corresponds the Rate of Dissociation.

## Data Availability

The data used to support the findings of this study are included in the article.

## Supplementary Materials

The following are available online at https://www.mdpi.com/1422-0067/22/4/1709/s1. Table S1. The binding strength detected in the conditioned medium. Figure S1. Negative control of cell staining. Cells were stained with a secondary antibody, Alexa flour568 anti-human antibody. Scale bars = 32 µm.
