# Supplementary material for: A Novel Artificially Humanized Anti-Cripto-1 Antibody Suppressing Cancer Cell Growth
Source: Int J Mol Sci. 2021 Feb 8;22(4):1709. doi: 10.3390/ijms22041709 (PMC7915030; doi:10.3390/ijms22041709)
Supplement: Supplementary file 1 [file ijms-22-01709-s001.pdf]

International Journal of
Molecular Sciences

Supplementary Materials:

Table S1. Table shows binding strength detected in the conditioned medium.

Clone No.	Response*2 x10-1	Kdis*3 x10-2/s
8	5.52	1.84
35	4.84	1.86
90	4.70	1.96
212	5.99	2.09
224	5.00	1.91
226	4.90	1.94
242	4.80	1.87
292	4.20	2.07
331	3.80	1.81
N.C.*1	-0.41	-

*1: conditioned medium of non-transformed ExpiCHO-S cells.

*2: response calculated from the time window entered in the Steady State Analysis section of OCTET system.

*3: kdis (1/s) value corresponds the rate of dissociation.

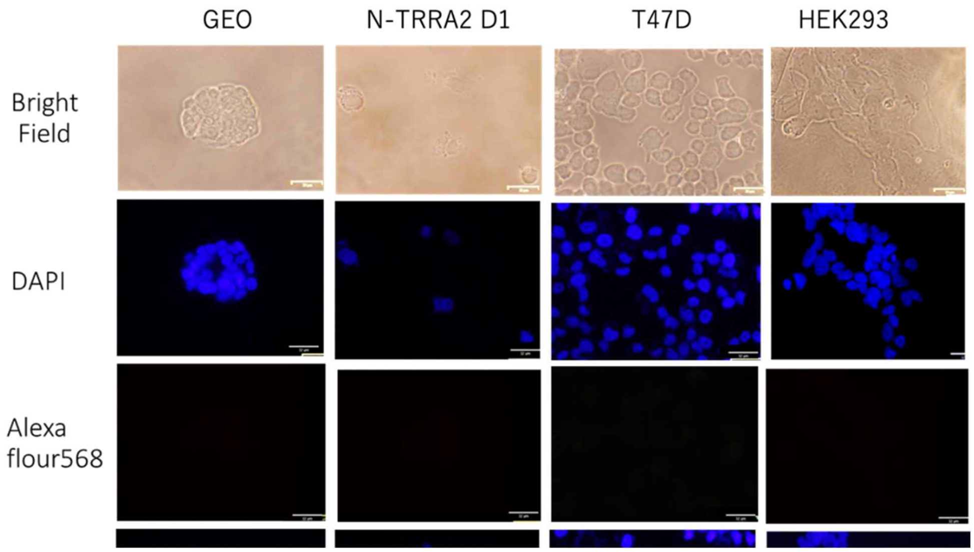

Figure S1. Negative control of cell staining**.** Cells were stained with a secondary antibody, Alexa flour568 anti-human antibody. Scale bars = 32 μm.
